# Novel immunobiologics for psoriasis

**DOI:** 10.4103/0253-7613.42300

**Published:** 2008-06

**Authors:** Nilanjan Ghosh, P.N. Singh, Vikas Kumar

**Affiliations:** Pharmacology Research Laboratory, Department of Pharmaceutics, Institute of Technology, Banaras Hindu University, Varanasi-221 005, India

**Keywords:** Antigen presenting cells, cytokines, helper T cells, psoriasis

## Abstract

Psoriasis is one of the most common human skin diseases and is considered to have key genetic contributions. It is characterized by excessive growth and aberrant differentiation of keratinocytes, but is reversible with appropriate therapy with the possibilities of recurrence. The trigger of the keratinocyte response is thought to be the activation of the cellular immune system with T cells, dendritic cells and various immune related cytokines and chemokines being implicated in pathogenesis. Immunosuppressants like cyclosporine and methotrexate were used earlier in the treatment of psoriasis, however their use was associated with severe adverse effects due to down regulation of immune system. The most recent advances in therapies for psoriasis target specific immune components of psoriasis and promise to have high therapeutic efficacy with low adverse effects. This review focuses on the novel therapies aimed to specifically modulate the dysregulated immune system with minimal adverse effects.

## Introduction

Psoriasis is an immune mediated genetic papulosquamous skin disorder which affects approximately 3% of world population. A substantial proportion of patients with psoriasis may develop a form of inflammatory arthritis known as psoriatic arthritis which contributes significantly to patient's disease burden with physical disability, pain and further reduction in quality of life.[1] Based on histological manifestation, in the past psoriasis was understood to be a disorder of keratinocyte hyper proliferation. However recently, with understanding of the etiology of the disease and the success of immunosuppressive therapies, the paradigm for understanding the disease has shifted towards T cell pathogenesis. Standard immunosuppressive therapies (like cyclosporine, methotrexate, hydroxyurea etc) which were used to treat psoriasis target different components of the immune response or block keratinocyte proliferation. However, these treatments are also associated with serious adverse effects like bone marrow depression, severe hepatotoxicity, renal dysfunction, gastrointestinal toxicity etc and may fail to induce long remission thus many patients elect not to undergo therapy.[2] In light of these pitfalls, much effort has been focused on immunobiologic agents, representing alternative approaches which are not only safe and effective but also allow longer remissions. Here we discuss the immunobiologics as novel therapeutic approaches for treatment of psoriasis.

### Types of psoriasis[3]

*Plaque psoriasis*: It is the most common type of psoriasis. The lesions appear as erythematous, scaly and often rounded plaques that are commonly found on elbows, knees, scalp and back.

*Guttate psoriasis*: It presents as small tear drop-shaped lesions on trunk and more proximal areas of extremities.

*Flexural psoriasis*: When lesions affect major skin folds particularly in genital regions, armpits and under the breasts, it is said to be flexural psoriasis or also inverse psoriasis. Scaly nature of lesions is absent in this type.

*Pustular psoriasis*: Patients with this type of psoriasis are often febrile with accompanying eruption of pustules over trunk and extremities.

*Psoriatic erythoderma*: It is characterized by total involvement of skin by active psoriasis.[3]

### Genetics of psoriasis

Psoriasis is a genetically heterogeneous disorder. Multiple genes are involved, and interactions with the environment are also implicated in its development. Three main genetic loci on chromosomes 17q, 4q, and 6p have been reported in genome scans.[4] HLA (Human Leukocyte Antigen) -Cw6 was first shown to be the HLA phenotype most strongly associated with psoriasis, the relative risk of those bearing the HLA-Cw6 phenotype to develop psoriasis being approximately 10-fold. Much of the genetic research has concentrated on the approximately 300-kb PSORS1 locus, near HLA-C on 6p21.3.[4] Other linkages of psoriasis have been made to 17q24-q25 (PSORS2), 4qter (PSORS3), 1q21 (PSORS4), and 3q21 (PSORS5) in different populations, although these linkages are not as reproducible as one with PSORS1.[5] Common variants in the SLC9A3R1/NAT9 region and a loss of a potential RUNX(Runt-related transcription factor 1) binding site have been described that could potentially affect regulation of the immune synapse.[6] There also has been a report of an association of psoriasis with the variant alleles of the lymphoid phosphatase PTPN22.[7]

### Pathogenesis of disease

Afferent arm: The afferent arm includes the physiological steps involved in the activation of naive T cells into active effector cells.[8]

Langerhan Cell (LC) and Dendritic cell (DC) changes: The initial changes involve the immature dendritic cells (DC) and Langerhan cells (LC) in the epidermis, which capture and internalize the antigen. This is then processed and presented on the cell surface, bound to class I and II major histocompatibility complex molecules. In addition, the LC also acquires a number of cell surface receptor markers such as CD80, CD86, CD40, CD83, and intercellular adhesion molecule (ICAM) 1. This  “mature” LC is now primed for stimulating the T cells in the draining lymph nodes.

**Figure 1:**
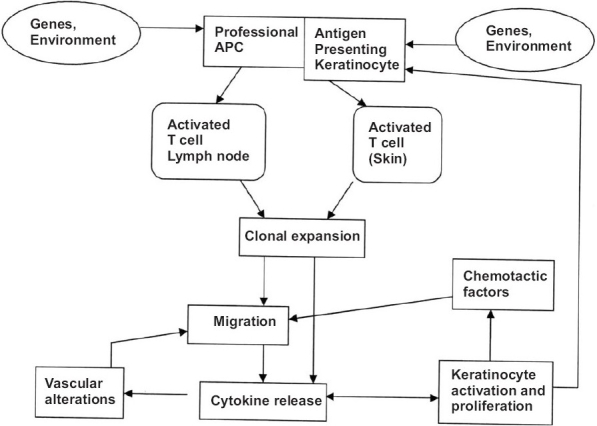
Schematic diagram of the pathophysiology of psoriasis

Subsequent T cell events lead to the activation of naive T cells in the lymph nodes into memory-effector (CD45RO) cells, differentiation and proliferation of these CD45^+^  cells.[9] Migration of activated T cells to sites of antigen excess in the skin, endothelial changes, expression of cell markers, and elaboration of various cytokines and adhesion molecules enhance this process and secretion of proinflammatory T1 cytokines by the effector T cells in the dermis and epidermis, with subsequent effects on keratinocyte proliferation.[10] The pathophysiology of psoriasis is shown in Figure 1.

*T-cell activation*  is a multi step process which involves:

*Binding: * Contact between the APC and the T cell is established by ICAM-1 and lymphocyte function-associated antigen (LFA) 3 on the APC with LFA-1 and CD2 on the T cells, respectively.

*Primary stimulation/signal 1: * The primary event is the recognition of the major histocompatibility complex-bound antigen by an appropriate T-cell receptor. Once a match occurs, T cell gets activated, with increase in the synthesis of mRNA for IL-2 and IL-2 receptor (CD25). Thus, the primary signal is always antigen-specific.[10]

*Co stimulation/signal 2: * Additional interactions also take place between the T cell and APC, together described as “accessory” or  “co stimulatory signals”. These are critical for optimal activation of the T lymphocyte. In the absence of co stimulation, the degree of responsiveness of the cell is very limited (anergy), or the cell itself may undergo apoptosis. These co stimulatory signals are not antigen-specific.

Co stimulatory interactions include: (i) CD80 and CD86 on the APC with CD28 on T cell, (ii) CD80 and CD86 on the APC with cutaneous T-lymphocyte antigen (CTLA) 4 on the T cell, which is an inhibitory signal, (iii) LFA3 with CD2, and (iv) CD40 and CD40 L.

**Figure 2:**
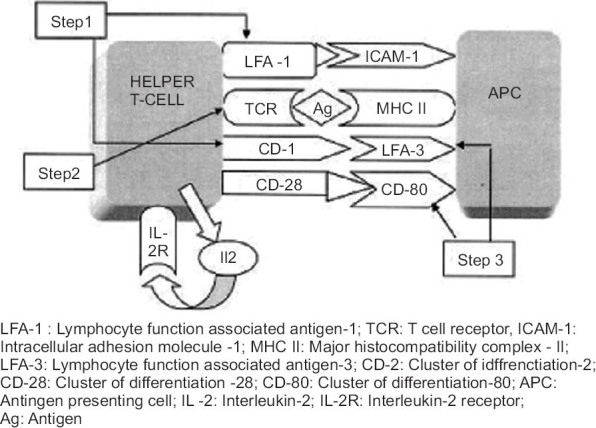
Steps involved in activation of Helper T cells by antigen presenting cells

IL-2 from T cells and IL-12 from mature LCs also bind to their receptors on the activated T cells. These reactions regulate transcription of cytokines such as interferon IFN-γ, TNF-α, IL-2, and granulocyte-macrophage colony-stimulating factor (GMCSF). These cytokines are responsible for differentiation, maturation, and proliferation (mitotic activity) of the T cells into memory-effector cells. The naive T cells are thus processed to mature into T_H_ 1 and type 1 cytotoxic T cell producing type 1 cytokines. Some of the T cells may also differentiate toward natural killer cell formation, which react with nonprotein antigens.[11] The steps involved in activation of helper T cells by antigen presenting cells are depicted in Figure 2. 

Efferent arm: The efferent arm includes the processes involved in migration of T cells to the site of antigen excess in the inflamed skin and their subsequent effects on keratinocytes. Migration of effector T cells to the site of antigen excess in the inflamed skin is the next step. T cells acquire a surface protein termed cutaneous lymphocyte-associated antigen. This is an adhesion molecule that mediates attachment of the T cell to the endothelial cells of the dermal vasculature (through E selectin and P selectin), with subsequent entry into the skin. The process also involves triggering of many chemokines and binding of integrins such as vascular cell adhesion molecule (VCAM), intercellular adhesion molecule (ICAM-1), and LFA-1.

*Effects of T cells on site:* Once in the dermis, the T_H_ 1 and type 1 cytotoxic T cells release high levels of IFN-γ and TNF-α. These induce ICAM-1, CD40, and major histocompatibility complex II proteins on the keratinocytes. Intraepidermal T cells trigger keratinocyte hyper proliferation, which accelerates epidermal growth occurring in the regenerative pathway.[12] It is likely that a cascade of cytokines, secreted by different cells in the local environment of the psoriatic plaque, play a role in the phenotypic responses in psoriasis. TNF-α has been found to increase type I vasoactive intestinal peptide (VIP) receptor mRNA in keratinocytes. Vasoactive intestinal peptide promotes keratinocyte proliferation and stimulates synthesis of proinflammatory cytokines such as IL-6, IL-8, and RANTES (regulated upon expression, normal T cell expressed and secreted).[13] These cytokines from intraepidermal T cells have been shown to be direct keratinocyte mitogens and thus could directly stimulate keratinocyte proliferation. TNF-α also increases plasminogen activator inhibitor type 2, a serine proteinase inhibitor, which is thought to protect cells from apoptosis.[14] The prevention of apoptosis by this or other mechanisms could lead to increased longevity of keratinocytes and consequently to a thickened epidermis. IFN-γ is also a trigger for epidermal hyperplasia when injected into skin.

The other proposed mechanisms for keratinocyte hyperplasia include wound reparative phenomenon that is triggered by intercellular disruption caused by T-cell entry into the epidermis. Mitogenic cytokines and receptors on keratinocytes such as epidermal growth factor (EGF), insulin like growth factor 1 and keratinocyte growth factor pathways are implicated in regenerative hyperplasia, and these would be stimulated as part of a wound repair response.[15] The other features of a psoriatic lesion, such as vascular proliferation and neutrophil infiltration are also caused by the action of the other cytokines such as vascular endothelial growth factor and IL-8 from keratinocytes on endothelium and polymorphonuclear leukocyte recruitment.[16]

### Development of immunobiologics

A significant challenge in the development of a biologic medication is the structural design of the drug. In other words, once a strategy has been identified and an appropriate target, such as a cell surface molecule or cytokine is chosen, how can a protein be made through recombinant DNA techniques to interact appropriately with that target? The new medication should have a number of characteristics: it should bind specifically to the target without interacting with other proteins, its binding should be sufficient for the desired effect, either deactivating or activating the target, it should persist long enough in the body to prevent the need for frequent dosing, and it should be immunologically as  “silent” as possible so as not to induce an immune response itself. The two main classes of biologics used include monoclonal antibodies and receptor-antibody fusion proteins. Monoclonal antibodies can be derived from genes that have different amount of murine sequences in the variable region. Chimeric antibodies have approximately 30% murine genes in the variable region, humanized antibodies have approximately 10% murine sequences and human antibodies are derived from human immunoglobulin genes.[17] Because humanized monoclonal antibodies are composed of more human genetic sequences than chimeric, and thus express less murine epitopes, humanized antibodies have a lower incidence of auto-reactivity due to the presence of auto antibodies. Meanwhile, fusion receptor-antibody proteins are split into two proteins. One portion has two sites that bind to proteins of interest, while the other part contains the Fc portion of human immunoglobulin, which brings stability to the protein structure.[18] The novel immunobiologics for psoriasis are summarized in Table 1.

**Table 1:** Novel immunobiologics for psoriasis

*Drug*	*Brand name*	*Manufacturer*	*Dose*	*Adverse effects*
Alefacept[38,43]	Amevive	Biogen Idec	15 mg IM once weakly for 12 weeks	Common: dizziness, nausea, chills, rhinitis, lymphopenia, and cough.
Efalizumab[24,25]	Raptiva	Genentech	0.7 mg/kg initial SC, then 1 mg/kg SC for nearly 12 weeks	Common: headache, fever, chills, nausea, myalgia, leukocytosis, lymphocytosis; severe: thrombocytopenia.
Infliximab[58,59]	Remicade	Centocor, Malvern	3-5 mg/kg IV at 0, 2, 6 and after every 8 weeks	Common: headache, nausea, diarrhea, injection site reaction, development of autoimmune and antichimeric antibodies; severe: opportunistic infections, malignancy.
Etanercept[65,66]	Enbrel	Amgen Inc, Thousand Oaks	50 mg SC for 12/24 weeks	Headache, fatigue, rhinitis, development of autoimmune antibodies; severe: opportunistic infections, malignancy, hematological reactions.
Adalimumab[61,62]	Humira	Abott laboratories	40 mg SC for 12 weeks	Headache, nausea, elevated triglycerides, cough, sinus congestion, injection site pain, and fatigue; severe:opportunistic infections, malignancy.
Daclizumab[31]	Zenepax	Roche pharmaceuticals	In clinical trials for psoriasis	Very rare
Basiliximab[33]	Simulect	Novartis	In clinical trials for psoriasis	Myalgia
CTLA4Ig/ Abatacept[26]	Orencia	Bristol-Myers Squibb	In clinical trials for psoriasis	Head ache, upper respiratory tract infections.
Denileukin difitox[35,36]	Ontak	Seragen	In clinical trials for psoriasis	Common: fever, asthenia, chills, myalgia, prurius, and vomiting.

### Evaluation of biologics

When evaluating the efficacy of biological agents, it is important to understand the standard measurement of efficacy in psoriasis treatment, the Psoriasis Area Severity Index (PASI). The PASI quantitates the extent and severity of skin involvement in different body regions as a score from 0 (no lesions) to 72 (severe disease). To gain FDA approval for the treatment of psoriasis, a biological agent must decrease the PASI by 75%. While such quantitation is an essential element in controlled clinical trials, many patients in practice may gain clinically significant benefit from biological treatment without achieving this degree of PASI improvement.[19]

### Action of immunobiologics

#### Signal 1 inhibition

Anti-CD4 antibody: OKTcdr4a. The underlying strategy behind OKTcdr4a (ORTHOCLONE), a humanized antihuman CD4 IgG4 monoclonal antibody derived from the murine OKT4A, is to prevent the generation of signal 1 in CD4+ cells.[20–22] In one small open label study, six patients with severe chronic plaque psoriasis were administered a total of three 1 mg/kg/day OKTcdr4a IV infusions every other day. After 4 weeks, patients experienced a mean reduction of 46% from baseline PASI values. One patient did not respond to treatment. Of the five responders, disease remission lasted 6 months in three patients and up to 1 year in one patient.

#### Signal 2 inhibition

Anti-CD11a antibody: Efalizumab: LFA-1, a heterodimer of CD11a and CD18, is a member of the β2-integrin family of cellular adhesion molecules found on T lymphocytes. It interacts with ICAM-1 on APC's to generate signal 2 and regulates T cell trafficking by interacting with the ICAM-1 molecules on endothelial cells and epithelial keratinocytes. Thus, blocking LFA-1 interactions has the dual effect of blocking activation as well as migratory activity of inflammatory T cells. As a unique subunit of LFA-1, CD11 represents a more selective target for immunosuppression than blocking CD18.[23] Efalizumab is a humanized monoclonal antibody against CD11a subunit, whose high efficacy has been demonstrated in a number of clinical studies. In a longterm clinical study, 50% of 228 patients with chronic plaque psoriasis achieved PASI 75 after 15 months of continuous efalizumab treatment, suggesting sustained efficacy with efalizumab.[24] Efalizumab was approved for treatment of psoriasis by FDA in 2003.[25] Administration of efalizumab induces a peripheral leukocytosis (predominantly of CD8+ memory cells), which is due to blockade of the LFA-1/ICAM-1 interaction between T cells and endothelial cells.

Anti-B7 antibodies: B7.1 (CD80) and B7.2 (CD86) are surface ligands APC's which bind to T cell CD28 to provide co stimulatory signals, as well as CTLA4 (CD152) to inhibit further T cell activation. As an inhibitor of the immune response, CTLA4 is expressed only on activated T cells and possesses a higher binding affinity for B7 than CD28.

### CTLA4Ig/Abatacept

CTLA4Ig/abatacept (BMS-188667) is a fusion protein composed of the extra cellular domain of CTLA4 and the Fc region of IgG4. This agent competitively binds the B7.1 and B7.2 molecules on the surface of APCs, thereby preventing their co stimulatory interaction with CD28 on naive T cells and thus interfering with T cell activation.[26] Expression of cell surface markers like DC-LAMP, B7 and CD40 on dendritic cells and CD40 and MHC II on lesional keratinocytes was also reduced.[27] In an open label, dose-escalation, multi-center study, 43 patients with stable psoriasis vulgaris were divided into eight groups and given IV infusions of 0.5, 1, 2, 4, 8, 16, 25 and 50 mg/kg of CTLA4Ig at days 1, 2, 16, and 29. By week 25, 46% of patients evaluated achieved a 50% or more improvement in Physicians Global Assessment scale (PGA) compared to 4% in the placebo group.[28]

### IDEC-114

IDEC-114 is a primatized monoclonal antibody that binds to the high-affinity CD80 receptor found on APCs and certain activated T cells. On the surface of activated T cells, CTLA4 (CD152 and CD28) is up-regulated and may then interact with CD80, leading to deactivation of the T cell. IDEC-114 blocks the CD28/CD80 co-stimulatory interaction which is an activation signal without affecting the CTLA4/CD80 interaction which is the deactivation signal.[27,28] In a phase I open label study, 24 patients with severe plaque psoriasis received one single IV infusion of 0.05, 1, 5, 10, or 15 mg/kg IDEC-114. Clinical response was assessed by PASI, PGA, and Psoriasis Severity Scale. On day 29, mean PASI score improvement from baseline of 7%, 4%, 4%, 25%, and 6% was observed in patients receiving 0.05, 0.25, 1, 10, and 15 mg/kg, respectively. No improvement in mean PASI scores for the 5-mg/kg group was observed. Eighty percent of the 10-mg/kg group and 60% of the 15-mg/kg group had PGA rating of fair or better. When scored by Psoriasis Severity Scale, 19%, 6%, 11%, 32%, and 17% improvements were observed in the 0.25, 1, 5, 10, and 15-mg/kg groups, respectively.[29]

### Targeting pathogenic T cells

Activated and memory T cells selectively up regulate characteristic surface receptors, which effect proliferative or activating functions. Specific biologics have been designed to block these surface markers and hence inhibit stimulatory signals to the potentially pathogenic T cells.

### Anti-IL-2R

IL-2R exists in three forms of varying affinity: α-CD25, β-CD122 and γ-CD132. Fully activated T cells increase expression of IL-2, which acts as a potent autocrine mitogenic stimulus.[30] This is accompanied by increased expression of the high-affinity form of its receptor IL-2R. Because IL-2R promotes T cell growth and proliferation, antibodies blocking its interaction with ligand will prevent clonal expansion of activated T cells.[30]

### Anti-CD25 antibodies

#### Daclizumab

Daclizumab is a humanized monoclonal antibody directed against CD25 (α-subunit), the subunit contributing to the high-affinity form of IL-2R. It has been FDA approved for acute renal transplant rejection and is currently under evaluation for the treatment of psoriasis.[31] In one multi-center study, 19 patients with moderate to severe plaque psoriasis received IV infusions of an initial 2-mg/kg dose followed by 1 mg/kg doses at weeks 2, 4, 8, and 12. At 8 weeks, patients who had initial PASI scores less than 36 exhibited a mean improvement of 30% thus suggesting that it might be effective treatment for patients with less severe disease.[31]

### Basiliximab

Basiliximab is a chimeric monoclonal antibody also directed against the CD25 subunit of IL-2. Although these anti-CD25 agents suggest efficacy as a potential therapy for psoriasis, combining treatment with IL-2 inhibitors may yield even better clinical outcomes. High quantities of IL-2 are still capable of activating T cells by cross-linking CD122 and CD132. Hence, it may be more ideal to combine treatment with cyclosporine, which inhibits IL-2 synthesis.[32,33]

### IL-2 fusion toxins: DAB (389) IL-2 (Denileukin Diftitox)

It is a fusion toxin constructed from the IL-2 gene and the enzymatically active ADP-ribosyltransferase domain of diphtheria toxin. The agent complexes with the IL-2 receptor and is endocytosed within the cell, whereupon the ADP-ribosyltransferase unit is cleaved and translocated to the cytosol. This blocks protein translation and induces cell apoptosis.[34] The FDA has already approved this agent for the treatment of CD25+ cutaneous T-cell lymphoma and is currently being evaluated for the treatment of psoriasis.[35] The most recent study has been a phase II multicenter, randomized controlled trial in which 35 patients with severe plaque psoriasis were placed on lower dosing regimens of 0.5, 1.5, or 5 mg/kg of DAB IL-2 administered by IV infusion for 3 consecutive days every other week for 8 weeks. Eight patients experienced a 50% decrease in baseline PASI and 51% of all patients experienced a decrease in PGA by at least one grade at some point in the 8-week treatment period and the dosing regimen provided a dose-dependent response.[36]

### Anti-CD2 antibodies

CD2 is expressed on the surface of T cells and natural killer cells. Its interaction with LFA-3 on the surface of APCs provides an accessory co stimulatory signal involved in full T cell activation. Because CD2 expression is up regulated in both CD4+ and CD8+ memory T cells (CD45RO+), agents that block CD2 will selectively inhibit the activation and proliferation of the pathogenic memory T cells. Such selective targeting is thought to represent a greater advantage over other strategies, in that nonpathogenic naive T cells are spared and left to participate in normal host immune responses.[37]

### Alefacept

It is a human fusion protein that consists of extra cellular domain of LFA-3 fused with CH2 and CH3 domain of IgG1 (Fc region). It competitively binds to CD2 cells on T cells to block its activation. It was also found that bound T cells are targeted to cytolytic destruction because the Fc region interacted with the FcγRIII on natural killer cells.[38] Alefacept was the first biologic to receive FDA approval for treatment of psoriasis. Because the memory T cells contain the highest number of CD2 on their surface membrane, alefacept is effective in selectively inhibiting T cell function. Despite the decrease in memory T cells, alefacept does not inhibit primary or secondary defense responses to foreign antigens. This may be explained by the fact that alefacept does not affect the naive T cell population.[39,40] A range of inflammatory genes such as IFN-γ, STAT1, MIG (CXCL9) and iNOS are reduced.[41,42] As the first biologic to be approved by the Food and Drug Administration (FDA) for moderate to severe psoriasis, alefacept has helped 70% of patients reach at least PASI 50, a clinically significant endpoint for psoriatic patients that indicates 50% improvement in PASI scores in a randomized controlled trial.[43]

### MEDI-507

MEDI-507 (Siplizumab), humanized IgG1 monoclonal antibody also directed against CD2 is currently in phase II clinical trials. The results of clinical studies suggested that multiple courses of IV administered MEDI-507 represent a well-tolerated treatment for psoriasis.[44] In an open label, dose escalation study, 39 patients with moderate to severe psoriasis were injected with 0.1-0.7 mg MEDI-507 SC every week for 12 weeks. Reductions in PASI scores were observed at all doses, but were more pronounced at higher doses.[45]

### Immune deviation

After naive T cells are activated, they differentiate and proliferate along a T1 or T2 cell lineage, depending on the cytokine environment. In psoriasis, the cytokine expression profile is selectively skewed toward a T1 (i.e., IL-2, TNF-α, IL-12, IFN-γ) and away from a T2 (i.e., IL-2, IL-5, IL-6, IL-10, IL-13) pattern. Altering the balance of T1 and T2 cells and restoring its proper ratio is thought to represent another therapeutic approach. Addition of recombinant type 2 cytokines, which not only has the effect of directly increasing T2 cytokine levels, but also inhibiting the differentiation of naive T cells into T1 cells, has been tried in the treatment of psoriasis.

### Tenovil (rhIL-10)

The anti-inflammatory and immunosuppressive T2 cytokine IL-10 has been found to be under expressed in psoriatic lesions. It inhibits the secretion of IFN-γ from T_H_ 1 cells and IL-12 from APC's. A number of studies have suggested the efficacy of recombinant human IL-10 (rhIL-10) (Tenovil) in improving psoriasis disease activity and also achieving longterm disease remission.[46,47] In a phase II, open label study 15 patients with moderate to severe psoriasis vulgaris were administered 4 mg/kg SC injections of rhIL-10 daily for 6 weeks. After 4 weeks of treatment, patients exhibited a mean decrease of 50% from baseline PASI; after 6 weeks of treatment, patients exhibited a mean decrease of 59% from baseline PASI. The results of this study provided evidence of the therapeutic potential of rhIL-10.[7]

### Oprelvekin (rhIL-11)

rhIL-11 (Oprelvekin) is a multifunctional cytokine that has been previously demonstrated to exhibit anti-inflammatory properties *in vitro*  and *in vivo*. Its interaction with macrophages leads to decreased production of type 1 cytokines, while its interaction with T cells leads to differentiation along the T1 lineage rhIL-11 has been FDA approved for the treatment of chemotherapy-induced thrombocytopenia and is in phase II clinical trials for the treatment of psoriasis.[48,49] In an open label, dose-escalation trial, 12 patients with moderate to severe psoriasis vulgaris were administered 2.5 or 5.0 mg/kg SC injections of rhIL-11 daily for 8 weeks. During the 8 weeks of post treatment, 30-80% decrease from mean baseline PASI in 11 of 12 patients were observed.[49]

### rhuIL-4

IL-4 is a type 2 cytokine which causes selective differentiation of T_H_ 2 cells. This property is propagated to be therapeutically important in psoriasis.[50] The first study involving *in vivo*  rhuIL-4 administration in humans was a dose-escalation study involving 22 patients with moderate to severe psoriasis vulgaris rhuIL-4 was injected SC at doses of 0.05, 0.1, 0.2, or 0.5 mg/kg three times daily, 5 days a week, for 6 weeks. After 3 weeks of treatment, the dosage was increased to the next level in each group except the 0.5-mg/kg group. Within 6 weeks, all patients experienced PASI reductions, with 19 exhibiting a greater than 50% reduction from baseline PASI. Groups receiving 0.2 or 0.5 mg/kg exhibited more reduction in PASI than other lower dose treatment groups.[50]

### Cytokine inhibitors

Proinflammatory cytokines secreted by activated T cells have also been targeted in the treatment of psoriasis. TNF-α is one such proinflammatory cytokine highly elevated in active plaques, serum, and synovial fluids of patients with psoriasis, with levels correlating to disease severity.[51] Tumor necrosis factor-α contributes to psoriasis pathogenesis in a variety of ways. It is a potent mitogenic stimulus that acts on epidermal keratinocytes to induce hyper proliferation, an inducer of epidermal VEGF expression (responsible for much of the inflammatory vascular changes associated with psoriasis), and an inducer of other proinflammatory cytokines such as IL-1, IL-6, IL-8, and TNF-α. Currently, TNF-α inhibitors have been the subject of much interest because of their demonstrated efficacy in disease improvement compared to other cytokine inhibitors.

### Infliximab

Infliximab, a chimeric monoclonal anti-TNF-α IgG antibody, binds soluble and transmembrane TNF-α. It is currently FDA approved for the treatment of rheumatoid arthritis and Crohn's disease, but remains in phase III clinical trials for the treatment of psoriasis.[52] Many studies have also demonstrated its efficacy in psoriatic arthritis. One of the features that distinguish infliximab from other anti-TNF-α biologics such as Etanercept is its ability to fix complement and lyse target cells.[53] Exacerbation of tuberculosis enteritis,[54] development of opportunistic infections such as Listeriosis,[55] serum sickness reactions,[56] multiple sclerosis flares in patients with preexisting disease are observed.[57] Absolute contraindications include CHF, active tuberculosis and history of multiple sclerosis and malignancy including lymphoma.[58] A study with 378 patients with moderate-to-severe plaque psoriasis randomized to 5 mg/kg infliximab or placebo infusions at weeks 0, 2 and 6, and every 8 weeks afterwards have been completed. By week 10, approximately 80% of infliximab-treated patients reached PASI 75, and 57% of these same patients reached PASI 90. At week 50, 61% of these patients were at PASI 75, while 45% achieved PASI 90.[59]

### Adalimumab

Adalimumab is a human recombinant IgG1 anti TNF-α monoclonal antibody, which is similar to infliximab, binds soluble and transmembrane TNF-α. In addition, adalimumab increases the number of epidermal Langerhan cells in psoriatic plaques. This finding suggests that not only Langerhan cells may have an anti-inflammatory role in psoriasis but also that TNF-α inhibition may restore Langerhan cell migration to epidermis.[60] It is currently FDA approved for the treatment of rheumatoid arthritis. Like infliximab, reports of tuberculosis, opportunistic infections, and malignancy exists with adalimumab as well. As with all TNF-α inhibitors, caution should be exercised when using these agents in patients with predisposing conditions.[61] A multi-center, randomized, doubleblind, placebo-controlled study involving 147 patients with moderate-to-severe psoriasis compared subcutaneous injections of 40 mg of adalimumab weekly, 40 mg of adalimumab every other week and placebo. At week 12 of treatment, 80% of patients receiving the weekly adalimumab treatments, and 53% of those receiving adalimumab every other week reached PASI 75. This response was sustained up to 60 weeks of therapy.[62]

### Etanercept

Etanercept is a fully human fusion protein consisting of two TNF receptors joined to the Fc region of IgG1. It binds soluble TNF-α and TNF-β, thus preventing either molecule from activating cell surface TNF receptors, TNF-R1 and TNF-R2. By decreasing NF-KB transcriptional activity in psoriatic skin, etanercept decreases epidermal thickness.[63] Etanercept reduces expression of cytokines and chemokines, such as IL-8 and MIP-3α (CCL20) in psoriatic lesions, leading to subsequent decrease in T cells, DC's and keratinocyte hyperplasia.[64] Structural differences also render it more unstable compared to infliximab. Etanercept is currently FDA approved for the treatment of juvenile rheumatoid and rheumatoid arthritis, Crohn's disease, ankylosing spondylitis, psoriatic arthritis and psoriasis.[65] A randomized, multi-center, double-blind controlled study of 112 patients with chronic stable plaque psoriasis taking either etanercept 25 mg SC or placebo was performed. While 30% of the etanercept group versus 2% of the placebo group reached PASI 75 after 12 weeks of treatment, 56% of the etanercept-treated patients versus 5% of the placebo-treated patients achieved PASI 75 after 24 weeks of treatment.[66]

### Toxicity of immunobiologics

#### Allergic reaction and antibody formation

They are mostly seen with TNF-α blockers. Mild transient injection site reactions comprising of erythema, edema and bruising are noted with etanercept in 10-20% of cases. They resolve spontaneously in 2-3 days and tend to occur in the first month of therapy. Antibodies to etanercept may develop in 6% of patients.[67] With infliximab, infusion reaction occurs during or within 1-2 hours of treatment and may affect up to 20% of all the patients treated. It may rarely result in anaphylactic shock.[68] Acute flu-like symptoms including headache, chills, fever, nausea and myalgia may occur within 48h after administration of the first two doses of efalizumab.[69]

#### Infections

Reactivation of tuberculosis may occur on treatment with anti-TNF-α agents, as TNF-α plays a key role in host defense against mycobacterial infection, particularly in granuloma formation and inhibition of mycobacterial dissemination. The risk of tuberculosis with infliximab has been estimated to be six times, in patients in trials for rheumatoid arthritis and Crohn's disease, than that of untreated patients.[54] Most patients were also receiving one or more immunosuppressive agents, which might have contributed to reactivation and dissemination of tuberculosis. However, no cases of tuberculosis have been reported in clinical trials of either infliximab or etanercept in psoriasis, possibly due to minimal number of patients treated and probably, by monotherapy. Other serious infections reported with etanercept include sepsis secondary to *Listeria monocytogenes* and *Histoplasma capsulatum*.[55] Severe disseminated opportunistic infections have been reported in the HIV positive patients.

#### Neurological disease

TNF blockers are associated with the development of or worsening of demyelinating disease. Worsening of multiple sclerosis and demyelination has been reported with infliximab.[68]

#### Cardiovascular disease

Worsening of congestive cardiac failure with TNF-α blockers is reported to occur. Patients with pre-existing heart failure (New York Heart Association class III and IV) failed to show benefit with TNF-α blockers and carried an excess mortality rate with high-dose infliximab.[57]

#### Antinuclear antibodies and lupus like syndrome

Antinuclear antibodies and anti-dsDNA antibodies may develop during therapy with anti-TNF-α agents, but it is not associated with symptoms and signs of lupus in the majority.[58]

#### Hepatitis

Rare cases of severe hepatitis have been reported following infliximab therapy, with onset of symptoms or signs occurring from 2 weeks to more than a year after initiation of treatment. The infliximab treatment should be stopped in the event of jaundice and/ or marked elevations (>5 times upper limit of normal) in liver enzymes.[59]

#### Thrombocytopenia

It can occur with efalizumab and warrants discontinuation of therapy.[70]

## Conclusion

We are at the dawn of a new era of effective immune modifying therapeutics for psoriasis. Although, efficacy of many of these agents is unquestionable, the longterm consequences of their immunomodulatory effect are yet to be firmly established. Responses may also vary between individuals, perhaps because of polymorphism in genes. With greater understanding of cutaneous immunology and further knowledge uncovering the mechanisms controlling the differentiation of activated T cells during an immune response, new biologic immune modifiers with highly specific immune targeted therapeutics can be developed that should have a better longterm safety than the currently available therapeutic agents.
